# Review of Role of Surgery with Electroporation in Melanoma: Chemotherapy, Immunotherapy, and Gene Delivery

**DOI:** 10.3390/jcm13133828

**Published:** 2024-06-29

**Authors:** M. Usman Ahmad, Allyson Walsh, Amanda Kirane

**Affiliations:** 1 Department of Surgery, Stanford University, Stanford, CA 94305, USA; akirane@stanford.edu; 2 Moores Cancer Center, University of California San Diego Health, San Diego, CA 92103, USA; alwalsh@ucsd.edu

**Keywords:** electroporation, melanoma, surgery, gene therapy, immunotherapy, chemotherapy, electrochemotherapy

## Abstract

Electroporation with chemotherapy (ECT) is currently offered as a treatment in Europe for locoregional or metastatic melanoma with cutaneous lesions. However, the role of surgery and other forms of electroporation in melanoma requires further evaluation. Two reviewers used two databases to conduct a literature search and review, and 51 publications related to electroporation with chemotherapy, immunotherapy, or gene delivery were found. ECT appears to be effective in reducing tumor burden for surgical resection, replacing surgical intervention with evidence of complete regression in some lesions, and inducing both local and systemic immune effects. These immune effects are pronounced when ECT is combined with immunotherapy, with a statistically significant improvement in overall survival (OS). Other forms of electroporation, such as those using calcium chloride, an IL-12 plasmid, and vaccination, require further study. However, IL-12 plasmid electroporation may be inferior to ECT based on the evidence available. Furthermore, irradiation of the tumor prior to ECT treatment is negatively correlated with local response. Access to ECT is restricted in the US and requires further evaluation. More randomized controlled trials of ECT and electroporation treatment in locoregional melanoma are recommended.

## 1. Introduction

In 2020, 325 thousand individuals were diagnosed with melanoma globally, with the number of new cases expected increase to 510 thousand per year by 2040 [1]. Mortality was highest in Australia, Europe, North America, and Northern Europe [1]. In the United States (US), the incidence of melanoma by stage is 82.1% for local disease, 10.4% for regional disease, and 3.7% for distant disease, with the remainder unstaged [2]. The five-year survival rates are 93.4%, 52.5%, and 16.9% for these patients, respectively [2]. In a Danish cohort, the incidence was similar, but the 10-year survival rates were reported by stage, with melanoma-specific deaths at 1.4–5.7% in Stage I, 13.6–28.5% in Stage II, 13–58.6% in Stage III, and 44.5% in Stage IV at 10 years [3]. These calculations were done in a modern cohort in a well-defined system showing a higher rate of mortality than derived from US data. This finding is supported by a second, modern, German cohort with melanoma-specific death up to 10% in Stage I and 35% in Stage II at 10 years, providing additional recent evidence of a worse-than-expected survival at earlier stages [4]. Overall, this points to a need for improved management of earlier-stage melanoma (Stage I–III) despite improved modern treatment inventions for locoregional melanoma.

Current treatment options for locoregional melanoma include surgery, systemic therapies before or after surgery, intralesional treatment with virus (T-VEC) or immunotherapy, isolated limb perfusion, local ablation with laser treatment, imiquimod, and radiation [5,6,7]. Adjuvant therapy for surgically resectable disease is currently approved by the US FDA for Stage II or greater melanoma with immunotherapy [8]. Neoadjuvant or pre-surgical treatment is recommended by NCCN guidelines for Stage III and above melanoma [6,9]. Neoadjuvant treatment with chemotherapy, immunotherapy, or radiation is considered to improve outcomes for several other cancers including breast, colon, pancreas, lung, gastric, and gastro-esophageal cancers [10,11,12,13,14,15]. However, evidence is evolving on the benefits of neoadjuvant therapy for melanoma [8,16].

**Figure 1:**
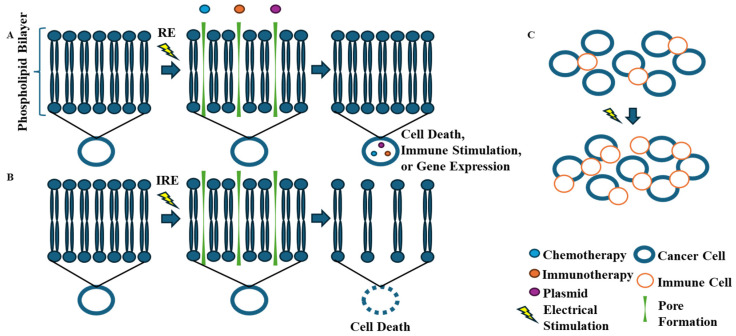
Mechanism of electroporation. (**A**) Reversible electroporation (RE) induces reversible change in cell membrane allowing for improved drug delivery or plasmid transfer. (**B**) Irreversible electroporation (IRE) induces non-reversible pore formation which leads to immediate cell death. (**C**) Other electrical fields and stimulation parameters also induce localized effects in tumor microenvironment such as immune cell stimulation and polarization.

Electroporation is a technique used to improve the permeability of cells via the application of electric fields to transfer DNA or genes and improve the effectiveness of systemic treatment [17]. Mechanistically, electroporation can be delivered to induce reversible and irreversible effects on the cell membranes, allowing for pore formation. Reversible electroporation (RE) allows for intracellular delivery of chemotherapy, immunotherapy, or gene transfer, as depicted in Figure 1A [17,18,19]. RE increases the permeability of cells to various chemotherapy types but has the largest effect (5000-fold) on bleomycin delivery, allowing for much lower doses than are required as a stand-alone agent [20]. Irreversible electroporation (IRE) utilizes different field parameters to induce a non-reversible change that immediately leads to cell death, as shown in Figure 1B [19]. There are also various other stimulation parameters that have been shown to improve immune cell infiltration and macrophage polarization in tumor models, as shown in Figure 1C [21]. Electrochemotherapy (ECT) is a technique utilized with systemic chemotherapy in addition to local electroporation for tumors in order to improve therapy efficacy. The specifications of treatment by tumor size, chemotherapy dose, and follow-up have been standardized since 2006 and were updated in 2018 and published as the European Standard Operating Procedures for Electrochemotherapy (ESOPE) [22]. This has been evaluated in melanoma for Stage III/IV disease, showing dramatic effects on local tumor control with an overall response rate of 77.6–81.5% [23,24,25]. However, only the European Society for Medical Oncology (ESMO) currently lists ECT as a potential treatment for locoregional melanoma, while the US and Australian guidelines do not yet have an application for the treatment modality [7]. Furthermore, electroporation may enhance the effects of local tumor responses when used with gene transfer, immunotherapy, and vaccines, and may have distant or abscopal effects, which may have significant systemic effects [17,26,27]. 

There is a continued increase in the clinical use of electroporation and/or ECT for advanced melanoma patients, with a validated need for more aggressive treatment for locoregional melanoma at risk of recurrence and progression. This review will summarize all published clinical data on the use of electroporation techniques on melanoma in order to determine its potential as a treatment tool for locoregional disease in the setting of surgical downstaging or surgical alternatives.

## 2. Methods

**Figure 2:**
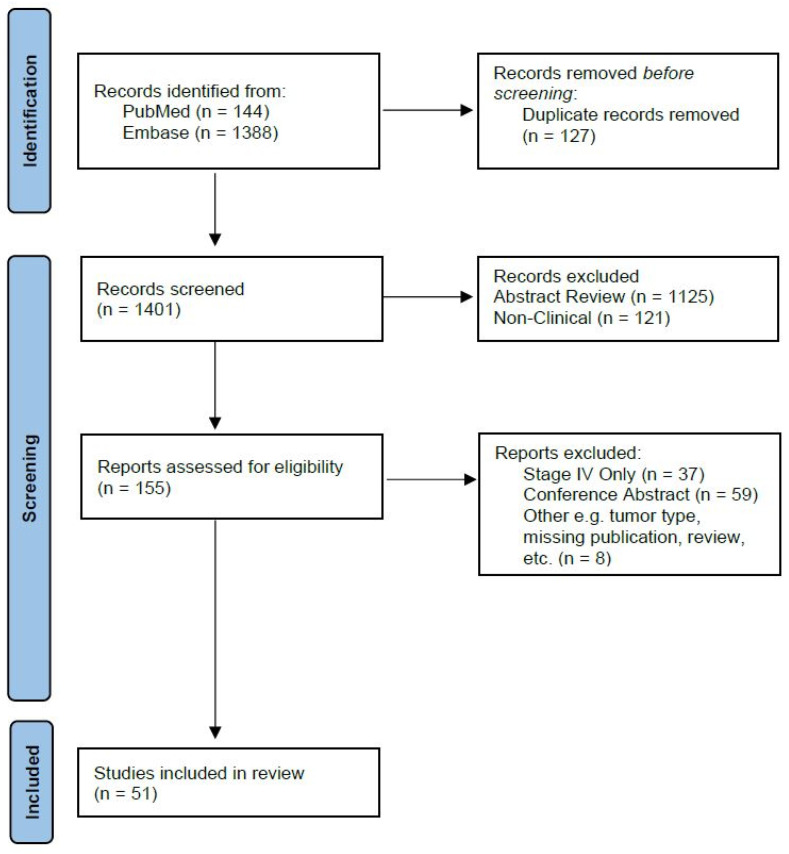
Adapted PRISMA flow diagram of search strategy including inclusion and exclusion criteria.

A literature search was conducted for case reports, clinical trials, and observational/retrospective reviews using PubMed and Embase for all languages in January 2024. Two reviewers searched for any articles containing the terms “melanoma” AND “electroporation”. Inclusion criteria included any human trial on melanoma using any type of electroporation. Exclusion criteria included conference abstract-only results, studies where only Stage IV melanoma patients were included, missing publications, and review articles or meta-analyses. Our review is focused on patients where surgery offers survival or therapeutic benefit, thus studies with only Stage IV patients were excluded. However, due to the valuable data from studies where both Stage III and Stage IV patients were included, these publications were not excluded and are further detailed in separate tables. The search strategy with the inclusion and exclusion criteria are summarized in an adapted PRISMA flow diagram in Figure 2 [28].

## 3. Results

Overall, 1401 records were screened with only 51 meeting the inclusion criteria.

### 3.1. Electrochemotherapy

#### 3.1.1. Stage III Melanoma

In Stage III melanoma, 14 case reports, clinical trials, and investigator-initiated studies evaluated ECT on Stage I–III melanoma, with 93% of studies using bleomycin and 50% of studies downstaging or debulking the tumor for locoregional treatment. The overall response (OR) rates ranged from 53–100%, and the complete response (CR) rates ranged from 75–100%, as depicted in Table 1 [21,22,23,24,25,26,27,28,29,30,31,32,33,34,35]. When evaluating publications including any surgery (biopsy or downstaging), there were four studies with five patients allowing for downstaging for surgical resection, confirmation of increased cytotoxic T-cell infiltration, and proof of tumor cell destruction. Rols et al. examined the effect of intravenous bleomycin dosed at 10 mg/m^2^ with four pulses of electroporation at 1000 V/cm under general anesthesia or neuroleptanalgesia, achieving a partial response (PR) considered at 50% tumor reduction and an OR in 90% of patients [29]. Rodriguez-Cuevas et al. used calculated doses of intralesional bleomycin and altered the parameters of stimulation to six pulses at 1300 V/cm and a frequency of 1 Hz under lidocaine injection on an outpatient basis, achieving a similar OR but improved CR rate of 23% when compared to Rols et al. [30]. Kubota et al. utilized 0.1 units of intralesional bleomycin per 1 mm of lesion with eight pulses of 100 microseconds at 1000 V/cm in perineal melanoma, resulting in a CR to the tumor and no recurrence for 2.5 years, avoiding the need for surgery [31]. Snoj et al. performed four sessions of ECT on extensive locoregional melanoma using intravenous bleomycin at 15,000 IU/m^2^ with eight pulses of 1300 V/cm over 100 microseconds, resulting in the regression of nodules and no systemic metastases for 9 months [33]. Quaglino et al. utilized 15 mg/m^2^ of intravenous bleomycin with square-wave pulses ranging from 1–5000 hertz for 20 min under general anesthesia with a CR rate of 58% [34]. In a follow-up study, the immune response was examined utilizing biopsies under the same treatment parameters, and results showed increased CD8 infiltration and lower (<10%) or negative FoxP3 staining in patients that did not develop visceral metastases [35]. Mozzillo et al. performed downstaging of a cheek melanoma on a patient using ECT with two treatments of eight to twelve pulses with 15,000 IU/m^2^ of intravenous bleomycin, allowing for conservative resection of the tumor (3.5 cm to 1.3 cm, no residual melanoma) [36]. Kendler et al. utilized intralesional bleomycin with the Cliniporator device over five treatments for two melanoma patients; both patients proceeded with stable disease (SD) at 6 weeks, but eventual progression was observed [37]. Carrera et al. conducted ECT in two patients with desmoplastic melanoma of the nose not amenable to surgery using intravenous bleomycin and up to two courses of treatment, eliciting CRs in both patients [38]. Bigi et al. utilized 15,000 IU/m^2^ of intravenous bleomycin with eight pulses of 1000 V/cm over 100 microseconds for 20 min, with CR of melanoma confirmed with biopsy [39]. Gallagher et al. downstaged tumors for resection and completed ECT using intravenous bleomycin and the Cliniporator device on two patients; patient 1 showed considerable debulking and response after failing pembrolizumab and T-VEC; patient 2, who did not respond to nivolumab, ipilimumab, or T-VEC prior ECT, showed tumor response after ECT was conducted [40]. Saad et al. utilized ECT for a patient not amenable to surgery or other systemic therapies after locoregional recurrence in the foot, and this resulted in a CR and no recurrence for 18 months [41]. In a case series of anorectal melanoma, Farricha et al. utilized intravenous bleomycin and 1000 V/cm, avoiding a morbid surgical procedure; along with local control, 62.5% were alive at 2 years and 50% had no evidence of disease [42]. Snoj et al. conducted cisplatin-based ECT on a patient with anorectal melanoma, allowing for sphincter preserving resection with no local recurrence at 14 months with the addition of radiation [32]. Overall, there appear to be differences in the route of administration of bleomycin (intralesional or intravenous) and stimulation parameters that may affect the local tumor response with CR rates varying from 9% to 58% between studies. Due to the location and complexity of some lesions, it should also be noted that the procedure may need to be conducted under general anesthesia, regional anesthesia, or local anesthesia, as indicated. The complexity of this technique requires multidisciplinary collaboration of expertise for solid tumor disease. Several case reports have also highlighted the value of surgical downstaging that ECT can offer in order to improve patient quality of life and outcome with locally progressive melanoma. This allows a patient to receive a significant benefit in cosmesis, function, morbidity, and potentially mortality. In addition, in certain cases, ECT may offer a tolerable, safe, and effective alternative to unproven treatments only available in clinical trials for some Stage I–III patients with unresponsive or unresectable disease. This is based on cases reported in patients who received available approved therapies, including systemic targeted therapy, immunotherapy, and oncolytic virus treatment, and achieved subsequent resolution after ECT treatment. 

#### 3.1.2. Stage III/IV Melanoma

In Stage III/IV melanoma, 22 case reports, clinical trials, and investigator-initiated studies evaluated ECT on Stage III/IV melanoma, with most using bleomycin, and 77% replacing surgery for locoregional treatment, achieving OR rates ranging from 50–100%, as depicted in Table 2 [36,37,38,39,40,41,42,43,44,45,46,47,48,49,50,51,52,53,54,55,56,57]. When comparing ECT to chemotherapy, there were three publications with 41 patients with a CR rate of 72–74% and 13–26%, and an OR rate of 78% and 38%, respectively. Heller et al. conducted ECT with intralesional bleomycin under local anesthesia with 99-microsecond pulses at 1300 V/cm at 1 hertz, with 90.9% of patients achieving a CR with confirmation of complete necrosis on biopsy [43]. Byrne et al. utilized local infiltration with bleomycin under local anesthesia with 100-microsecond pulses ranging between 560–1500 V, resulting in a CR rate of 72% compared to a CR rate of 26% using bleomycin alone [46]. Gandy et al. also used intralesional bleomycin with 100-microsecond pulses at 4 hertz at 600 V/cm, resulting in a 74% CR rate compared to a 13% CR rate with bleomycin alone [47]. Campana et al. evaluated patients using both intravenous or intralesional bleomycin with two electroporation parameters and found that 50% achieved a CR across multiple tumor types. There did not appear to be a difference based on the route of bleomycin administration [48]. Campana et al. also evaluated a series of melanoma tumors under the same parameters (n = 85), with 48% achieving a CR, which was associated with increasing electrode applications (*p* = 0.041) and cycles of treatment (*p* = 0.005) [49]. Caracò et al. treated melanoma patients with intravenous bleomycin at 15,000 IU/m^2^ using ESOPE, with 48.4% of patients achieving a CR and 21.7% being disease-free at 27.5 months after one session [50]. Gerlini et al. conducted ECT with intravenous bleomycin using Cliniporator specifications and found large shifts in HLA-DR+ cells and dendritic cells over 2 weeks post-treatment, proposing a mechanism for localized and abscopal responses [51]. Caracò et al. evaluated a second cohort of patients (n = 34) receiving ECT using intravenous bleomycin under ESOPE guidelines, and found that 48.3% achieved a CR [52]. Kreuter et al. evaluated a multicenter cohort of patients receiving ECT with intravenous bleomycin under ESOPE guidelines, and 20% of patients had a CR, which was the largest amongst tumor types [53]. Mir-Bonafé et al. evaluated patients treated with intravenous bleomycin under ESOPE ECT, with 27% achieving a CR and 49% experiencing disease stability [54]. Campana et al. evaluated another cohort using ECT with ESOPE guidelines and a lesion-based choice of intravenous or intralesional bleomycin, with 50% achieving a CR and a median overall survival (OS) of 34.6 months [55]. Di Gennaro et al. conducted ECT with intravenous bleomycin under standard protocol, achieving a 60% CR rate, with CD3+ cells increasing from day 0 to day 14 (65.33 vs. 84.38, *p* < 0.01) [56]. Kunte et al. evaluated a multi-institutional cohort of melanoma patients treated with ECT under ESOPE guidelines, using either intravenous or intralesional bleomycin, and found that 58% achieved a CR, with radiation negatively correlating with the response [57]. Al-Hadithy examined a cohort of patients in the UK undergoing ECT under ESOPE guidelines and intravenous bleomycin and found that 47% achieved a CR [58]. Rotunno et al. evaluated an Italian cohort with ECT conducted with intravenous or intralesional bleomycin under ESOPE guidelines and found that 58% achieved a CR, with previous radiation identified as a negative predictor of response [59]. Borgognoni et al. evaluated patients undergoing ECT with intravenous bleomycin and the Cliniporator device, finding CR rates of 61.3% for <3 cm lesions compared to 31.8% for >3 cm lesions [60]. Clover et al. evaluated a multi-national cohort of patients undergoing ECT under ESOPE guidelines, using intravenous or intralesional bleomycin, which achieved a 65% CR rate, with radiation again showing a negative correlation with response [61]. Simioni et al. evaluated ECT with intravenous bleomycin under ESOPE guidelines, which achieved a 69.2% CR rate for melanoma [62]. Campana et al. used a similar procedure for ECT on another cohort, achieving a 47% CR rate, preserving quality of life during treatment [63]. Zietek et al. reviewed patients at a single center with ECT using intravenous bleomycin and achieved 100% PR [64]. Serša et al. conducted two studies using ECT with intravenous cisplatin, comparing ECT to chemotherapy alone, with a 68% vs. 19% CR rate in the larger trial [44,45]. Several comparative trials have examined the benefit of using ECT vs. chemotherapy alone, with substantial differences in patient outcomes and local tumor resolution reported. This data highlights the significant value that electroporation provides, even in the case of using much older, generic chemotherapy such as bleomycin or cisplatin. CR of the local tumor is subject to various other factors such as tumor size, electric field, concentration of intralesional or intravenous chemotherapy, number of ECT cycles, and previous irradiation of the tumor. Overall, CR seems to improve with increased electroporation of the tumor cells to improve drug delivery with repeated cycles; however, this effect seems to be inhibited when the tumor is irradiated prior to ECT. Increased drug permeability seems to be the central factor in achieving a CR; however, the finding of a negative correlation to response with previous irradiation requires further study, the mechanism of which is currently unknown. In addition, ECT appears to have an immunologic effect on the tumor, confirmed by biopsy and analysis of tissue throughout the treatment course. Both lymphocytic and myeloid infiltration of the tumor increase after ECT treatment, showing a profound and complex change in the pro-inflammatory tumor micro-environment, which appears to allow adaptations in adaptive and innate immunity. 

### 3.2. Electrochemotherapy and Immunotherapy or Other Adjuvants

#### 3.2.1. Stage III Melanoma

In Stage III melanoma, four case reports and investigator-initiated studies evaluated ECT with adjuvants as definitive locoregional treatment, with CR rates of 80–100%, as depicted in Table 3 [65,66,67,68]. Only two case reports evaluated modern immunotherapy, e.g., nivolumab or ipilimumab, with a CR rate of 100% in both patients. Gehl et al. reported a patient with melanoma on a clinical trial receiving low-dose interleukin-2 and ECT with intravenous bleomycin, which resulted in nine nodules on the patient showing CR [65]. Hribernik et al. evaluated ECT using bleomycin or cisplatin in combination with interferon alpha, which resulted in a 100% CR rate with cisplatin and a 66% CR rate with bleomycin [66]. Quaresmini reported a case of nodular melanoma treated initially with intravenous bleomycin ECT, which resulted in recurrence. Subsequent treatment with nivolumab showed no response, but combination therapy induced spontaneous regression, along with immune-related adverse events; the patient is disease-free [67]. Morgese et al. reported the case of a patient with lentigo melanoma of the scalp with locoregional progression after resection and pembrolizumab; the patient received intravenous bleomycin ECT and ipilimumab, which resulted in complete regression of disease [68]. Overall, the sample sizes in combination therapy using ECT and immunotherapy are too small for reliable interpretation of effects on a heterogeneous patient population. However, the aforementioned reports provide evidence of the significant potential for improved patient responses, which warrants larger randomized controlled trials. This effect may be due to the fact that previous studies have shown a shift in the tumor immune microenvironment with ECT alone, which may improve the efficacy of currently available immunotherapies.

#### 3.2.2. Stage III/IV Melanoma

In Stage III/IV melanoma, five investigator-initiated studies and clinical trials evaluated ECT with adjuvants, with 80% of modern trials utilizing ECT as definitive locoregional treatment, with an ORR/OR of 40–77.8%, as depicted in Table 4 [69,70,71,72,73]. Plesnicar et al. evaluated five patients with ECT in combination with chemoimmunotherapy, reporting a 40% PR rate [69]. Serša et al. evaluated the effect of chemoimmunotherapy without and with electroporation in nine patients, reporting an 11% CR rate in both groups, with a 22% vs. 48% OR rate, and 4 vs. 21 weeks to progression [70]. Mozzillo et al. evaluated ECT with bleomycin and ipilimumab, reporting a 27% CR rate locally, an immune-related disease control rate (irDCR) of 60%, and an 86.2% survival rate at 12 months [71]. irDCR was defined as the sum of complete response, partial response, and stable disease, according to radiologic criteria at up to 3 months [71]. Heppt examined patients undergoing treatment with intravenous bleomycin and immunotherapy with ipilimumab or pembrolizumab, which resulted in a CR rate of 15.2% locally and a systemic response rate of 22.6% [72]. Campana et al. evaluated 130 patients and compared ECT with pembrolizumab vs. pembrolizumab alone, showing a benefit of combination therapy on OS RR 2.02 (95% CI 1.01–4.03, *p* = 0.046) [73]. The OS difference of ECT vs. pembrolizumab was not described in the results [73]. Earlier reports using combination immunotherapy with ECT had small sample sizes, inconsistent treatment regimens, and variable outcomes. However, these studies do show the potential of the additive benefit of immunotherapy in patients receiving ECT, with an improvement in systemic response. This effect of adding immunotherapy to ECT was more pronounced in the larger trial with Campana et al., involving 130 patients randomized to multiple treatment arms. They showed significant improvements in survival when using combination therapy when compared to using immunotherapy alone. Given the lack of current treatment options, the ease of use, affordability, efficacy, and low rate of adverse events, combination ECT + immunotherapy appears to be a safe and effective treatment option for patients requiring additional therapy and may be considered earlier in treatment. 

### 3.3. Electroporation and Calcium

In Stage III/IV melanoma, two clinical trials evaluated electroporation with calcium chloride vs. ECT with no statistically significant differences between treatment groups on treatment response as depicted in Table 5 [74,75]. Overall, the option to use calcium chloride instead of chemotherapy is attractive due to the potential of adverse events with traditional chemotherapy. Unfortunately, although the results are promising in showing non-inferiority in trials, the sample sizes are too small to provide enough evidence for conclusive decision-making for alternatives to chemotherapy in ECT. Further study is required.

### 3.4. Electroporation and Gene Delivery/Vaccine

In Stage III/IV melanoma, four clinical trials evaluated electroporation of IL-12 plasmids or melanoma antigens as depicted in Table 6 [76,77,78,79]. The OR/ORR did not appear to improve when treated with EP + IL-12 vs. EP + IL-12 + pembrolizumab across three clinical trials (53–64% vs. 48%) [76,77,78]. Electroporated vaccination with the melanoma antigen, pINGmuTyr, was conducted in a Phase I trial to assess the appropriate dose and response, and they found that measurements of CD8 tumor-infiltrating lymphocytes improved most in the 1.5 mg dosage group [79]. Local infiltration of immune cells of melanoma was assessed in three published trials using an electroporated IL-12 plasmid. Theoretically, IL-12 should produce a localized pro-inflammatory immune response, thereby allowing both localized and systemic responses due to changes in innate and subsequently adaptive immunity. Unfortunately, in Daud et al., the CR rate of injected lesions was 9%, which is much lower than the rate seen in ECT trials [76]. In Greaney et al., the CR rate was not reported by patient or by lesion, but there was a shift in immune cell infiltration, with increased CD3+ cells. However, PD-1+ cells decreased systemically, indicating a paradoxical change in local and systemic immune responses [77]. Given the low rates of CR in the previous study, it is likely that IL-12 alone did not induce as significant of an immune effect as ECT treatment. When combined with immunotherapy, IL-12 electroporation showed an ORR of 48% compared to 77.8% in the ECT + pembrolizumab trial, which shows the trajectory of paradoxical reaction in the local and systemic immune compartments [78]. When comparing rates of effectiveness against ECT treatment, IL-12 electroporation appears to be inferior. However, these results are limited by sample size. The study by Yuan et al. on electroporated antigen vaccination appears to be interesting but requires further study in order to determine its ability to improve treatment and survival in melanoma patients [79]. 

### 3.5. Ongoing Clinical Trials

Only two clinical trials are currently active or in progress comparing different ECT regimens and adjuvant pembrolizumab in Stage III/IV patients, as shown in Table 7. Unfortunately, at this time, planned clinical studies are limited to only Stage III/IV patients with small sample sizes. The largest potential benefit may be in early intervention for locoregional patients, where a localized stimulatory immune effect of ECT + immunotherapy may offer a better chance of reducing recurrence and improving OS. More trials for locoregional melanoma with combination therapy should be pursued in appropriately selected patients in order to continue providing evidence of the substantial potential improvement in cancer treatment.

## 4. Discussion

Due to recurrence and metastasis in locoregional melanoma, targeted adjuvant systemic therapy is already considered after surgical resection, including modern immunotherapy drugs. However, the field of ECT in melanoma has not been broadly applied to treatment globally despite promising results and low rates of adverse events. In the largest trial with Stage III melanoma using bleomycin ECT, eight of fifteen patients had a localized response associated with increased expression of CD8 lymphocytes [35]. Furthermore, in the largest trial of Stage III/IV patients using bleomycin-based ECT, the local tumor control rate was 78% at 1 year, 68% at 2 years, and 62% at 3 years [63]. In addition, health-related quality of life was similar to that of the general population in this cohort, highlighting the limited adverse events and patient discomfort due to treatment. Mechanistically, in a Stage III/IV melanoma population undergoing bleomycin-based ECT, Gerlini et al. highlighted the increase in CD1c+ dermal dendritic cells (dDCs) during treatment; day 0 (5.63%); day 7 (8.34%); day 14 (16.11%); *p* < 0.05 and *p* < 0.001 [51]. This effect may point to an additive benefit of bleomycin ECT in stimulating the immune response when combined with modern immunotherapy. Although not included in this review due to the exclusion criteria, the potential abscopal effect was pronounced in a patient case with Stage IV acral melanoma. The treatment involved using a combination of surgery, temozolomide, and ECT with bleomycin and calcium, and the patient’s lesions and lymph node metastases completely regressed, with pronounced vitiligo and recurrence at 17 months [80]. Patient reactions such as these are typically reserved for situations where immunotherapy is used, highlighting the local and systemic immune effect that ECT may have without using adjuvant immunotherapies. In addition, it is important to note that multiple trials show a negative correlation of local response with previous irradiation to the tumor, which should be addressed in future trial designs. The causal relationship of poor response after irradiation requires further investigation.

Combination treatments using ECT have not been evaluated extensively in Stage I-III melanoma, although there are case reports and small series with promising results. In a Phase II trial for Stage III/IV melanoma comparing bleomycin-based ECT + pembrolizumab vs. pembrolizumab, there was a significant improvement in local PFS (5.76 RR, CI 2.41–13.77, *p* < 0.001), systemic PFS (1.06 RR, CI 1.07–3.60, *p* = 0.030), and OS (2.02 RR, CI 1.01–4.03, *p* = 0.046) [73]. Interestingly, the dose of bleomycin may be reduced in ECT without reducing effectiveness [24]. In some settings chemotherapy using bleomycin has been replaced with calcium chloride. However, although there was no significant difference in local response, the studies have been too small to correlate clinically [74,75]. Given the possibility of reducing systemic adverse events and improving survival in Stage III/IV melanoma patients using a combination of ECT with bleomycin and immunotherapy, further study is warranted in randomized controlled trials with high-risk locoregional patients.

The use of electroporation in gene delivery has been an ongoing concept for several decades in the scientific community and is currently being studied for the treatment of melanoma in clinical trials. Three trials have been published showing the potential clinical efficacy of electroporated intra-tumoral delivery of IL-12 plasmids. IL-12 may be related to immune-stimulating effects of T and NK cells [77]. In a Phase II trial evaluating the combination of IL-12 electroporation with pembrolizumab in immunologically cold tumors, transcriptional analysis of the biopsies showed increased CD8 TILs (*p* = 0.027) and PD-L1 expression (*p* = 0.016) when comparing pre- to post-treatment [78]. Median OS was not reached in this study at 19.6 months and may need to exceed 36 months in order to show comparable value to the ECT + pembrolizumab trial [73,78]. Other electroporation techniques studied clinically in melanoma include a Phase I trial of vaccination using a melanoma tumor antigen. At this time, the dose-finding trial did find an increase in TILs, with median OS not reached at 40.9 months [79]. Importantly, this trial does have Stage II patients, which may affect the OS results. Compared to ECT, IL-12 electroporation appears to show inferior localized and systemic immune effects; however, due to a lack of comparative randomized trials, a conclusive recommendation of inferiority cannot be given.

At this time, only two clinical trials are registered using electroporation or ECT in melanoma, with an emphasis on extrapolating differences in chemotherapy regimens and combination treatments with pembrolizumab. Given the potential benefit of combination therapy on local response, systemic response, and survival, more studies are warranted. Electroporation and combination therapy appear to hold potential as a low-risk, high-benefit option for Stage II-III melanoma patients with a higher risk of recurrence. The choice of agent should reflect modern regimens and balance local and systemic toxicity. 

## 5. Conclusions

Electroporation of chemotherapy using bleomycin shows profound effectiveness in reducing local tumor burden, allowing for lesions to be surgically resectable in locoregional melanoma. However, in some cases, the tumor is eliminated, precluding the need for surgery for these patients. In addition, when combined with pembrolizumab, ECT with bleomycin shows an improvement in OS compared to immunotherapy alone, showing a current use case for patients who may need adjuvant treatment with a low rate of adverse effects. Access to ECT is restricted in the US and requires further evaluation. Importantly, radiation may reduce the effectiveness of ECT. Other therapies such as electroporation of calcium, IL-12 plasmids, and tumor antigens require further study.

## 6. Future Directions

ECT with bleomycin should be evaluated extensively in locoregional melanoma in combination with other treatments due to its low side effect profile, ease of application, low cost, and potential ability to improve the effectiveness of modern immunotherapies. Other forms of electroporated therapy also require further study in larger populations.

## Figures and Tables

**Table 1:** Electrochemotherapy in Stage III melanoma.

Author	Year	Type	Treatment	Drug	Timing of Surgery	# Patients	Stage	Outcome	Metric
Rols	2000	IIS	ECT	Bleomycin	Mixed	4	Stage III	OR	90%
Rodríguez-Cuevas	2001	Phase II	ECT	Bleomycin	_	2	Stage III	OR	84.50%
Kubota	2005	Case Report	ECT	Bleomycin	After	1	Stage III	RFS	2.5 years
Snoj	2007	Case Report	ECT	Bleomycin	_	1	Stage III	LPFS	9 months
Quaglino	2008	Phase II	ECT	Bleomycin	_	14	Stage III	OR	93%
Quaglino	2011	Phase II	ECT	Bleomycin	_	15	Stage III	OR	53%
Mozzillo	2012	Case Report	ECT	Bleomycin	After	1	Stage III	OR	100%
Kendler	2013	Phase II	ECT	Bleomycin	Mixed	2	Stage III	SD	100%
Carrera	2014	Case Series	ECT	Bleomycin	_	2	Stage II/III	CR	100%
Bigi	2016	Phase II	ECT	Bleomycin	After	2	Stage III	OR	100%
Gallagher	2020	Case Report	ECT	Bleomycin	Mixed	2	Stage III	CR	100%
Saad	2020	Case Report	ECT	Bleomycin	_	1	Stage III	OR	100%
Farricha	2021	Phase II	ECT	Bleomycin	_	8	Stage III	CR	75%
Snoj	2005	Case Report	ECT	Cisplatin	After	1	Stage III	RFS	14 months

ECT = electrochemotherapy; IIS = investigator-initiated studies; OR = overall response; CR = complete response; RFS = recurrence-free survival; LPFS = local progression-free survival; SD = stable disease.

**Table 2:** Electrochemotherapy in Stage III/IV melanoma.

Author	Year	Type	Treatment	Drug	Timing of Surgery	# Patients	Stage	Outcome	Metric
Heller	1998	IIS	ECT	Bleomycin	After	12	Stage III/IV	CR	89%
Byrne	2005	Phase II	ECT vs. CT	Bleomycin	Mixed	19	Stage III/IV	CR	72% vs. 26%
Gaudy	2006	IIS	ECT vs. CT	Bleomycin	_	12	Stage III/IV	CR	74% vs. 13%
Campana	2009	Phase II	ECT	Bleomycin	_	34	Stage III/IV	OR	96% (all tumors)
Campana	2012	Phase II	ECT	Bleomycin	_	85	Stage III/IV	OR	94%
Caracò	2013	Phase II	ECT	Bleomycin	_	60	Stage III/IV	OR	87%
Gerlini	2013	Phase II	ECT	Bleomycin	After	9	Stage III/IV	OR	100%
Caracò	2015	Phase II	ECT	Bleomycin	_	34	Stage III/IV	PR	38%
Kreuter	2015	Phase II	ECT	Bleomycin	_	20	Stage III/IV	OR	50%
Mir-Bonafé	2015	Case Series	ECT	Bleomycin	_	31	Stage III/IV	OR	72%
Campana	2016	Phase II	ECT	Bleomycin	Before	211	Stage III/IV	CR	38%
Di Gennaro	2016	Phase II	ECT	Bleomycin	After	10	Stage III/IV	CR	60%
Kunte	2017	Phase II	ECT	Bleomycin	_	151	Stage III/IV	CR	34%
Al-Hadithy	2018	Phase II	ECT	Bleomycin	_	26	Stage III/IV	CR	65%
Rotunno	2018	Phase II	ECT	Bleomycin	_	22	Stage III/IV	CR	58%
Borgognoni	2020	Phase II	ECT	Bleomycin	_	44	Stage III/IV	OR (< 3 cm)	84%
Clover	2020	Phase II	ECT	Bleomycin	_	283	Stage III/IV	OR	82%
Simioni	2020	Phase II	ECT	Bleomycin	_	13	Stage III/IV	LPFS	62%
Campana	2022	Phase II	ECT	Bleomycin	_	378	Stage III/IV	CR	47%
Zietek	2022	Phase II	ECT	Bleomycin	_	5	Stage III/IV	PR	100%
Serša	1998	IIS	ECT	Cisplatin	_	2	Stage III/IV	CR	100%
Serša	2000	Phase II	ECT vs. CT	Cisplatin	_	10	Stage III/IV	OR	78% vs. 38%

IIS = investigator-initiated studies; ECT = electrochemotherapy; CT = chemotherapy; OR = overall response; CR = complete response; PR = partial response; LPFS = local progression-free survival.

**Table 3:** Electrochemotherapy with adjuvants in Stage III melanoma.

Author	Year	Type	Treatment	Drug	Timing of Surgery	# Patients	Stage	Outcome	Metric
Gehl	2000	Phase II	ECT + I	IL-2	_	1	Stage III	CR	100%
Hribernik	2016	IIS	ECT + IFN	Bleomycin or Cisplatin + Interferon-a	_	5	Stage I-III	CR	80%
Quaresmini	2021	Case Report	ECT + I	Bleomycin + Nivolumab	_	1	Stage III	CR	100%
Morgese	2023	Case Report	ECT + I	Bleomycin + Ipilimumab	_	1	Stage III	CR	100%

IIS = investigator-initiated studies; ECT = electrochemotherapy; I = immunotherapy; IFN = interferon; CR = complete response.

**Table 4:** Electrochemotherapy with adjuvants in Stage III/IV melanoma.

Author	Year	Type	Treatment	Drug	Timing of Surgery	# Patients	Stage	Outcome	Metric
Plesnicar	1994	IIS	ECT + I	HLI + Lomustine + Vinblastine + Bleomycin + Cisplatin +/− Tamoxifen	Before	5	Stage III/IV	OR	40%
Serša	2000	IIS	ECT + I	Cisplatin + Vinblastine + Lomustine + Interferon-a2b	_	9	Stage III/IV	OR	48% vs. 22%
Mozzillo	2015	Phase II	ECT + I	Bleomycin + Ipilimumab	_	15	Stage III/IV	ORR local	67%
Heppt	2016	Phase II	ECT + I	Bleomycin + Ipilimumab or Nivolumab or Pembrolizumab	_	33	Stage III/IV	ORR	67%
Campana	2021	Phase II	ECT vs. ECT + I vs. I	Bleomycin + Pembrolizumab	_	130	Stage III/IV	OR	80.5% vs. 77.8% vs. 38.6%

IIS = investigator-initiated studies; ECT = electrochemotherapy; I = immunotherapy; HLI = human leukocyte interferon; OR = overall response; ORR = overall response rate.

**Table 5:** Electroporation with calcium in Stage III/IV melanoma.

Author	Year	Type	Treatment	Drug	Timing of Surgery	# Patients	Stage	Outcome	Metric
Falk	2018	Phase II	EP + Calcium vs. ECT	Calcium Chloride or Bleomycin	_	1	Stage III	CR	100% vs. 100%
Ágoston	2020	Phase II	EP + Calcium vs. ECT	Calcium Chloride or Bleomycin	_	6	Stage III/IV	CR (all tumors)	22% vs. 40%

ECT = electrochemotherapy; EP = electroporation; CR = complete response.

**Table 6:** Electroporation with IL-12 plasmid/vaccine in Stage III/IV melanoma.

Author	Year	Type	Treatment	Drug	Timing of Surgery	# Patients	Stage	Outcome	Metric
Daud	2008	Phase I	EP + IL-12	IL-12 plasmid	After	24	Stage III/IV	OR	53%
Greaney	2020	Phase II	EP + IL-12	IL-12 plasmid	_	28	Stage III/IV	OR	64%
Algazi	2020	Phase II	EP + IL-12 + I	IL-12 plasmid + Pembrolizumab	_	23	Stage III/IV	ORR	48%
Yuan	2013	Phase I	EP + Vaccine	pINGmuTyr	_	24	Stage II/III/IV	CD8 TILs	40% in 1.5 mg group

EP = electroporation; I = immunotherapy; OR = overall response; ORR = overall response rate; TILs = tumor-infiltrating lymphocytes.

**Table 7:** Active clinical trials.

Name	NCT	Country	n	Design	Type
_	NCT06388252	Slovenia	15	ECT + bleomycin vs. ECT + cisplatin	Stage III/IV
_	NCT03448666	Italy	53	ECT + bleomycin + pembrolizumab	Stage III/IV

ECT = electrochemotherapy.

## Data Availability

Not applicable.
